# Comparison of background characteristics and neuropathology findings between medico-legal autopsy cases with traumatic axonal injury, vascular axonal injury, or absence of axonal injury in β-amyloid precursor protein stain

**DOI:** 10.1007/s00414-025-03415-3

**Published:** 2025-01-21

**Authors:** Gaia Narayan, Petteri Oura

**Affiliations:** 1 Department of Forensic Medicine, University of Helsinki, P.O. Box 21, Helsinki, FI-00014 Finland; 2 Forensic Medicine Unit, Finnish Institute for Health and Welfare, P.O. Box 30, Helsinki, FI-00271 Finland

**Keywords:** Traumatic brain injury, Forensic pathology, Axonal damage, Postmortem

## Abstract

In forensic neuropathology, the β-amyloid precursor protein (β-APP) immunostain is used to diagnose axonal injury (AI). The two most common aetiologies are traumatic (TAI) and ischaemic (vascular; VAI). We aimed to identify background characteristics and neuropathology findings that are suggestive of TAI, VAI, or no AI in neuropathologically examined medico-legal autopsy cases. The dataset comprised 166 cases from Finland over the period 2016—2023. The diagnosis of AI was based on β-APP stain (TAI, VAI, or no AI). Data on background characteristics and neuropathology findings were collected from cause-of-death investigation documents. Prevalence ratios were calculated for each variable to enable comparisons between the AI categories. The sample were 71.7% males; median age was 41 years (range 0—96). There were 26 cases with TAI, 44 with VAI, and 96 with no AI. The variables that showed statistical significance and had at least two-fold prevalence among TAI cases compared to VAI cases were: a documented recent injury; and presence of any extracranial/cranial/intracranial injury (including subdural haemorrhage [SDH], subarachnoid haemorrhage [SAH], intracerebral/ventricular haemorrhage [ICVH], or contusion) in autopsy or neuropathology. Correspondingly, variables indicating TAI over no AI were: a documented recent injury; postinjury survival ≥ 24 h; and presence of any extracranial/cranial/intracranial injury (including SDH, SAH, ICVH, contusion), herniation, or infarction in autopsy or neuropathology. Postinjury survival < 30 min was identified as an indicator of no AI over TAI. Finally, variables indicating VAI over no AI were: postinjury survival ≥ 24 h; lack of external injury to the head; and presence of SDH, brain oedema, herniation, or infarction in autopsy or neuropathology. In conclusion, we report several differences in characteristics and findings between cases diagnosed with TAI, VAI, and no AI. Our findings may help estimate the likelihood and potential aetiology of AI based on background characteristics and other neuropathology findings.

## Introduction

Given that the brain shows signs of inflammation after an insult, the presence of microglia cells was previously used to diagnose axonal injury (AI) [1], followed by silver stains [2]. In present-day forensic neuropathology, AI is identified by immunohistochemical β-amyloid precursor protein (β-APP) staining of axons proximal to damage [3]. In general, β-APP stains damaged axons in distinct patterns, depending on whether a traumatic shearing injury or ischaemic complications are involved [4–6]. Traumatic AI (TAI) is characterized by mechanical trauma resulting in shearing and distortion of axons in white matter [3] and was first defined by a three-tier grade of severity by Adams et al. [7]. Vascular AI (VAI) is a secondary resultant of complications of traumatic impact and hypoxia-ischemia; it is commonly linked to a raised intracranial pressure (ICP) [8].

Although β-APP stain has proven valuable to the diagnosis of AI, additional information is often useful and sometimes imperative for estimating the potential aetiology of AI. As such, attempts to establish background characteristics and neuropathology findings that help estimate the likelihood of AI and differentiate between TAI, VAI, and absence of AI are warranted.

The aim of this study was to identify factors characteristic of TAI, VAI, and absence of AI among medico-legal autopsy cases referred to a neuropathological examination and β-APP stain. It was hypothesized that cases involving recent head injuries would be associated with TAI; cases involving raised ICP and hypoxia-ischaemia would be associated with VAI; and very rapid deaths as well as those caused by processes that do generally not involve the brain would be associated with absence of AI.

## Materials and methods

### Material

According to the Act on the Investigation of the Cause of Death 1973/459, the Finnish police are required to investigate deaths that entail a possibility of homicide, suicide, accident, medical or surgical adverse event, occupational disease, as well as sudden and unexpected deaths. In most cases, the police order a medico-legal autopsy to be performed by a forensic pathologist in the Forensic Medicine Unit, Finnish Institute for Health and Welfare. A medico-legal autopsy is performed in approximately 15% of all deaths in Finland [9, 10]. Approximately 1 in 40 medico-legal autopsies involves a full neuropathological examination of a formalin-fixed brain performed by a board-certified neuropathologist [11].

The Helsinki office of the Forensic Medicine Unit covers Southern Finland and the capital metropolitan region, totaling approximately 3000 medico-legal autopsies annually. An electronic information system was adopted in 2016 as a comprehensive storage for medico-legal cause-of-death investigation documents. For the purposes of this study, a member of the research group accessed the documents of the cases that fulfilled the following inclusion criteria:

- A medico-legal autopsy performed at the Helsinki office in 2016—2023.
- The general autopsy supplemented with a full neuropathological examination performed by a consultant neuropathologist.
- β-APP staining performed in the neuropathological examination to diagnose AI.

The data collection covered all cases that fulfilled the inclusion criteria; there were no exclusions. The study was performed in accordance with the Declaration of Helsinki and national legislation on medical research. As the study was retrospective and based on register-based data only, ethical approval was not required. A research permit was obtained from the institutional data permit authority of the Finnish Institute for Health and Welfare (THL/1802/6.02.00/2023).

### Axonal injury status in β-APP stain

**Fig. 1:**
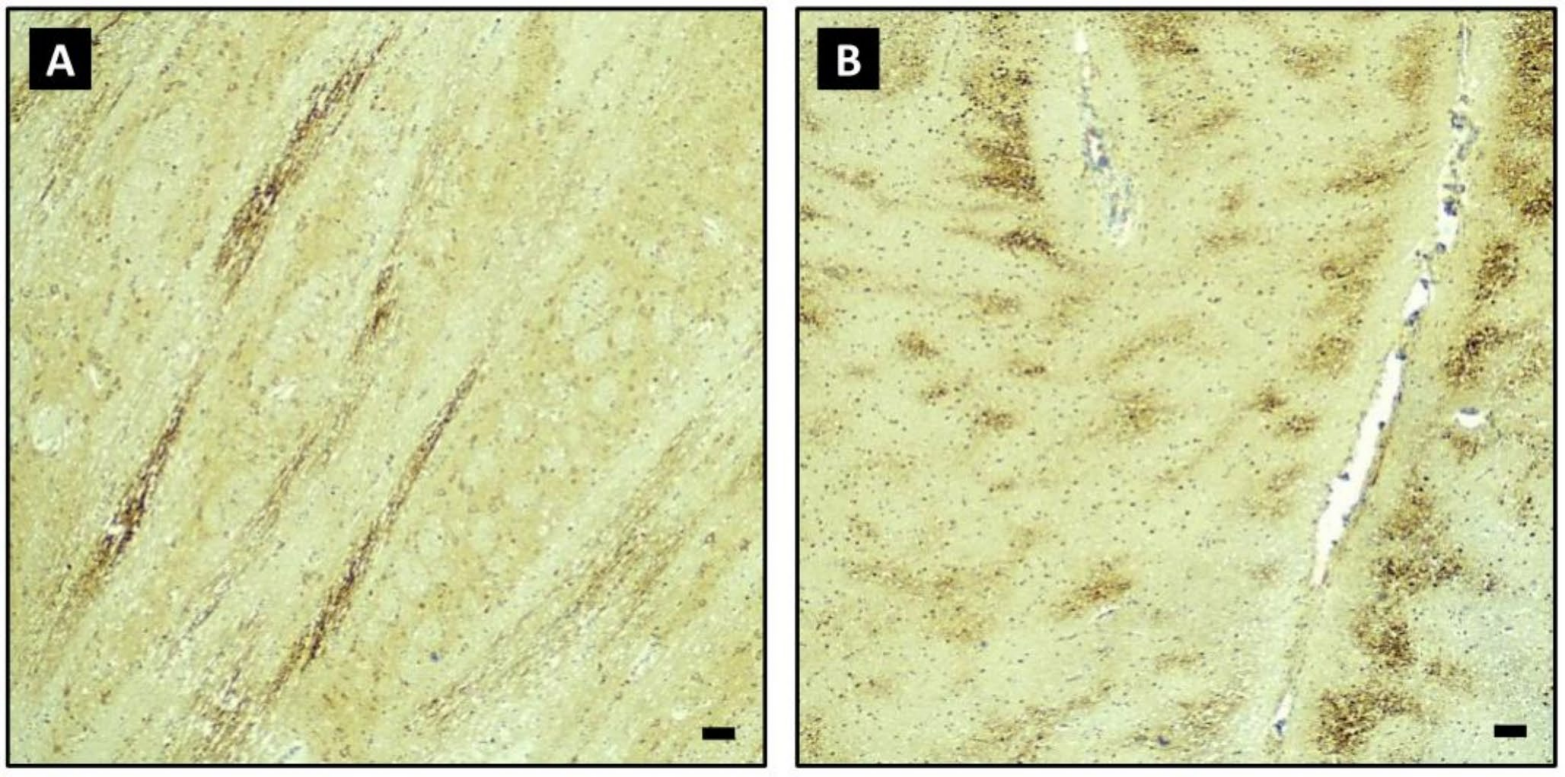
Sample micrographs demonstrating a typical staining pattern in traumatic axonal injury (**A**) and vascular axonal injury (**B**) in β-amyloid precursor protein stain. Both samples involve the pons, 40x magnification. Scale bars equal 0.1 mm

The consultant neuropathologist’s laboratory is part of a large, public, tertiary-level pathology department. A comprehensive neuropathological examination of the formaldehyde-fixed brain (minimum of two weeks) was performed by a board-certified neuropathologist. The examination included β-APP-stained samples (Anti-APP A4 Antibody, MAB348) of the corpus callosum, internal capsule, and brainstem, as a minimum standard. The aetiology of AI was estimated on the basis of the β-APP staining pattern. In brief, scattered β-APP-positive axons involving single white matter tracts are considered characteristic of TAI, whereas linear and geographical patterns of positive axons are regarded to represent VAI [2]. Figure 1 illustrates the typical staining patterns consistent with TAI and VAI.

The neuropathologist’s report was used to classify cases into the following AI categories:

1. Staining pattern suggestive of TAI (“TAI” group).
2. Staining pattern suggestive of VAI (“VAI” group).
3. No staining positivity suggestive of AI (“No AI” group).

Cases were classified as TAI or VAI regardless of whether the finding was focal, multifocal, or diffuse. To make categorizations mutually exclusive, each case was classified into one category only. Importantly, it was taken into consideration that TAI and VAI were expected to co-occur quite often in traumatic cases [4, 8]. If a case was reported to have a TAI pattern in any of its samples, it was classified into the TAI group even if secondary VAI was also reported. As such, the TAI group comprised cases with “pure TAI” and co-occurring TAI + VAI, whereas the VAI group only comprised cases with “pure VAI”. This decision was based on the fact that the main goal of a forensic pathologist is to distinguish traumatic cases (TAI with or without VAI) from non-traumatic ones (pure VAI).

For TAI and VAI cases, the location of AI was recorded (brainstem/internal capsule/corpus callosum/other/unspecified; yes/no for each). In TAI cases, also the diagnosis and grading of diffuse TAI (dTAI) was recorded according to Adams et al. [7].

### Background characteristics

The medico-legal autopsy order of the police was used to record the sex (male/female) and age of the decedent (years). Autopsy order and medical records were used to extract medical history regarding the central nervous system (CNS) (underlying diseases, surgical procedures, and remote injuries). Underlying conditions of the CNS were categorized in a data-driven manner (epilepsy or history of seizures/dementia or neurodegenerative disease/stroke or transient ischaemic attack/neoplasm of the CNS/hydrocephalus/other).

The autopsy order and medical records were also queried for a documented recent injury or asphyxic event (yes/no for each). In case a recent injury was documented, its general circumstance (fall/traffic accident/assault/other) and postinjury survival (< 30 min/30 min–23 h/≥ 24 h/unknown) were recorded. Postinjury survival was estimated as accurately as possible on the basis of the aforementioned documents.

The manner and primary cause of death were recorded from the death certificate issued by a forensic pathologist. The manner of death was recorded as disease/accident/homicide/other, and the primary causes of death were categorized in a data-driven manner (head or neck injury/stroke/cardiovascular disease/intoxication/asphyxiation/sudden infant death syndrome/other). Postmortem interval (days) was calculated by taking the difference between the known or estimated time of death and autopsy date.

### Autopsy and neuropathology findings

The general medico-legal autopsy report was used to record the following variables: fresh brain weight (g) of the decedent, presence of external injury to the head (laceration, stab wound, bruise, traumatic swelling), scalp haemorrhage, and fracture of the cranium, facial bones, or cervical spine (yes/no for each).

The presence of acute traumatic intracranial haemorrhages (epidural, subdural, subarachnoid, intracerebral/ventricular) were recorded from both the general autopsy and neuropathology reports (yes/no for each). The general autopsy report was used as the main source for epidural and subdural haemorrhage, and the neuropathology report for subarachnoid and intracerebral/ventricular haemorrhage. Contusions were documented from neuropathology reports.

The neuropathology report was also used to record the following variables (yes/no for each): brain oedema (macroscopic or microscopic), herniation, hypoxic-ischaemic neuronal injury (pyknosis and/or eosinophilia in haematoxylin-eosin stain; mostly mild and terminal), cerebral atrophy (cortical or central), vermal atrophy, neuropathological changes warranting a neurodegenerative disease diagnosis, atherosclerosis in the circle of Willis, arteriolosclerosis, cerebral amyloid angiopathy, and brain infarction (micro, lacunar, or major). All findings were recorded as reported by the neuropathologist, regardless of severity.

### Statistical analysis

The statistical analysis was performed in SPSS version 27 (IBM, Armonk, NY, USA). For continuous variables, medians were calculated and reported with interquartile ranges (IQRs). For categorical variables, prevalences were calculated for the three AI groups (TAI, VAI, no AI) and presented as percentages (%) with frequencies (n).

Comparisons between the three AI groups were based on categorical variables. The statistical differences in prevalences between the three AI groups were analyzed using the Chi square test and Fisher-Freeman-Halton Exact Test, as applicable. In order to offer the reader a better sense of effect sizes, pairwise head-to-head comparisons were performed on the basis of prevalence ratios (e.g., prevalence among TAI cases divided by prevalence among VAI cases). Statistical and clinical relevance was defined as follows: a statistically significant difference between groups (*P* < 0.05) combined with a prevalence ratio of > 2 or < 0.5, which indicates more than a two-fold difference in prevalence between groups. Prevalence ratios were illustrated on a logarithmic scale using graphs created in Excel version 2005 (Microsoft, Redmond, WA, USA).

## Results

The sample comprised 166 neuropathologically examined medico-legal autopsy cases, of whom 71.7% were male. The median age of the decedents was 41 years (IQR 16—62, full range 0—96) with a median postmortem interval of 6 days (IQR 3—9). The median brain mass was 1427 g (IQR 1262—1534).

Table 1 presents the characteristics of AI findings among the sample. A total of 26 cases were classified into the TAI group, 44 cases into the VAI group, and 96 cases into the no AI group. Of the TAI group, 7/26 cases (26.9%) had also VAI findings; these were considered secondary to trauma. Both TAI and VAI findings were most often observed in the corpus callosum (84.6% and 68.2%, respectively), followed by the internal capsules (61.5% and 59.1%) and brainstem (61.5% and 56.8%).

**Table 1:** Characteristics of axonal injury (AI) findings among the AI groups

	TAI (*n* = 26)	VAI (*n* = 44)	No AI (*n* = 96)
Location of TAI			
Brainstem	61.5 (16)	0.0 (0)	0.0 (0)
Internal capsule	61.5 (16)	0.0 (0)	0.0 (0)
Corpus callosum	84.6 (22)	0.0 (0)	0.0 (0)
Other	7.7 (2)	0.0 (0)	0.0 (0)
Unspecified	3.8 (1)	0.0 (0)	0.0 (0)
Location of VAI*			
Brainstem	11.5 (3)*	56.8 (25)	0.0 (0)
Internal capsule	11.5 (3)*	59.1 (26)	0.0 (0)
Corpus callosum	26.9 (7)*	68.2 (30)	0.0 (0)
Other	3.8 (1)*	9.1 (4)	0.0 (0)
Unspecified	0.0 (0)*	13.6 (6)	0.0 (0)
Grading of TAI**			
Focal or multifocal	23.1 (6)	-	-
Diffuse, grade 1	50.0 (13)	-	-
Diffuse, grade 2	15.4 (4)	-	-
Diffuse, grade 3	11.5 (3)	-	-

*In the TAI group, VAI was considered secondary to trauma

**According to Adams et al. [7]

Values are percentages (%) with frequencies (n)

TAI = Traumatic axonal injury, VAI = Vascular axonal injury

Table 2 presents a summary of underlying CNS conditions, primary causes of death, and manners of death among the AI groups. An underlying CNS condition was present in 42.2% of the sample; epilepsy or seizure history and dementia or neurodegenerative disease were the most frequent conditions. Among the TAI group, accident (50.0%) and homicide (26.9%) were the most common manners of death, while most deaths were certified as natural among the VAI group (65.9%) and no AI group (51.0%).

**Table 2:** Commonest underlying conditions of the central nervous system (CNS), causes of death, and manners of death among the axonal injury (AI) groups

	TAI (*n* = 26)	VAI (*n* = 44)	No AI (*n* = 96)
Underlying conditions of the CNS			
Any underlying condition	30.8 (8)	43.2 (19)	44.8 (43)
Epilepsy or history of seizures	11.5 (3)	13.6 (6)	26.0 (25)
Dementia or neurodegenerative disease	11.5 (3)	18.2 (8)	7.3 (7)
Stroke or transient ischemic attack	7.7 (2)	9.1 (4)	8.3 (8)
Neoplasm of the CNS	0.0 (0)	0.0 (0)	3.1 (3)
Hydrocephalus	0.0 (0)	0.0 (0)	2.1 (2)
Other	3.8 (1)	9.1 (4)	12.5 (12)
Primary cause of death			
Head or neck injury	50.0 (13)	6.8 (3)	10.4 (10)
Stroke	0.0 (0)	18.2 (8)	2.1 (2)
Cardiovascular disease	0.0 (0)	9.1 (4)	18.8 (18)
Intoxication	15.4 (4)	18.2 (8)	7.3 (7)
Asphyxiation	3.8 (1)	0.0 (0)	12.5 (12)
Sudden infant death syndrome	0.0 (0)	0.0 (0)	15.6 (15)
Other	30.8 (8)	47.7 (21)	33.3 (32)
Manner of death			
Disease	11.5 (3)	65.9 (29)	51.0 (49)
Accident	50.0 (13)	9.1 (4)	15.6 (15)
Homicide	26.9 (7)	9.1 (4)	11.5 (11)
Other	11.5 (3)	15.9 (7)	21.9 (21)

Values are percentages (%) with frequencies (n)

TAI = Traumatic axonal injury, VAI = Vascular axonal injury

**Fig. 2:**
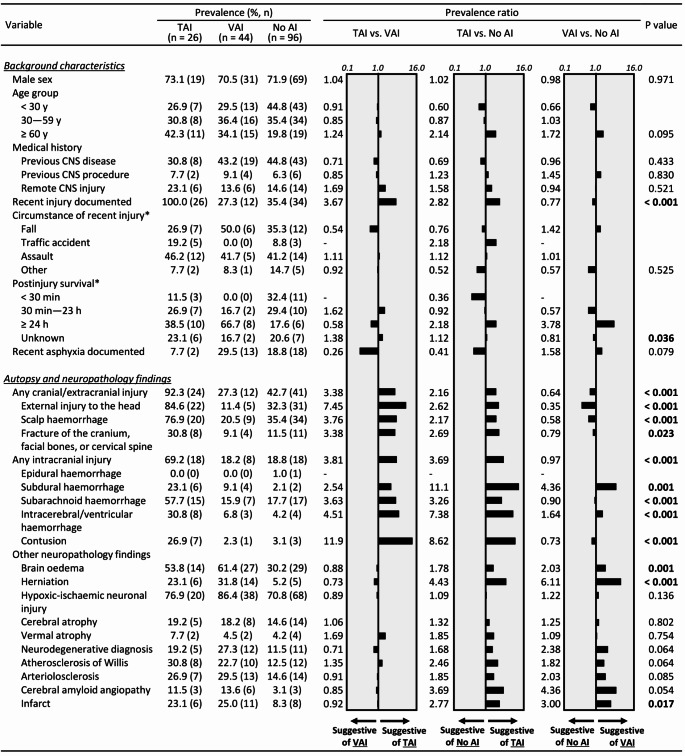
Comparison of background characteristics and autopsy and neuropathology findings relative to axonal injury (AI) status in β-APP stain. Prevalences are presented as percentages (%) and frequencies (n); in the two variables marked with an asterisk (*), the prevalences are calculated in relation to the set of cases that had a recent injury. Prevalence ratios are illustrated on a logarithmic scale. P values are obtained from Chi square test or Fisher-Freeman-Halton Exact Test, as applicable. β-APP = β-amyloid precursor protein, CNS = Central nervous system, TAI = Traumatic axonal injury, VAI = Vascular axonal injury

Figure 2 presents a comparison of background characteristics as well as autopsy and neuropathology findings between the AI groups. Below we cite the variables that are considered both statistically and clinically relevant, i.e., show a statistically significant difference between the AI categories and have a prevalence ratio of > 2 or < 0.5, indicating more than two-fold differences in prevalence between the categories.

First, the characteristics indicative of TAI over VAI were: a documented recent injury; presence of any cranial/extracranial injury, including external injury to the head, scalp haemorrhage, or fracture of the cranium, facial bones, or cervical spine; and presence of any intracranial injury, including subdural haemorrhage, subarachnoid haemorrhage, intracerebral/ventricular haemorrhage, or contusion in autopsy or neuropathology (Fig. 2).

Second, the characteristics indicative of TAI over the absence of AI were: a documented recent injury; postinjury survival of ≥ 24 h; presence of any cranial/extracranial injury, including external injury to the head, scalp haemorrhage, or fracture of the cranium, facial bones, or cervical spine; presence of any intracranial injury, including subdural haemorrhage, subarachnoid haemorrhage, intracerebral/ventricular haemorrhage, or contusion; and presence of herniation or infarction in autopsy or neuropathology (Fig. 2). In contrast, postinjury survival of < 30 min was identified as an indicator of no AI over TAI (Fig. 2).

Third, the characteristics indicative of VAI over the absence of AI were: postinjury survival of ≥ 24 h; lack of external injury to the head; presence of subdural haemorrhage; and presence of brain oedema, herniation, or infarction in autopsy or neuropathology (Fig. 2).

## Discussion

This study of 166 neuropathologically examined medico-legal autopsy cases identified background characteristics and neuropathological findings that differed between cases diagnosed with TAI, VAI, and absence of AI. The characteristics that were indicative of TAI over VAI, and TAI over the absence of AI, were suggestive of traumatic injury (e.g., a documented recent injury and presence of extracranial, cranial, or intracranial injuries). In contrast, the characteristics that indicated VAI over the absence of AI were suggestive of increased ICP and hypoxia-ischaemia (e.g., brain oedema, herniation, subdural haemorrhage, and infarction; often with the absence of external injuries to the head). These findings were mostly in line with our initial hypotheses.

As for background characteristics, a documented recent injury was indicative of TAI over both VAI and no AI. This was not surprising given the traumatic origin of axonal damage in TAI. Falls from a height, traffic accidents, and impacts of blunt trauma have been considered potentially characteristic circumstances for TAI due to intracranial shearing as an acceleration-deceleration mechanism related to the sudden movements of the head [8, 12, 13]. However, our data did not show statistically significant differences in the prevalence of injury circumstances between the AI groups. We speculate that this may be due to low statistical power and lack of detailed information on injury mechanisms in our dataset (e.g., the “falls” category included both simple falls or falls from a height).

Long postinjury survival (≥ 24 h) was found to be suggestive of both TAI and VAI over no AI. In contrast, short postinjury survival (< 30 min) was found to be indicative of the absence of AI over TAI. These findings were expected, as a minimum survival of approximately 30 min has conventionally been considered a prerequisite for diagnostic levels of β-APP immunopositivity to cumulate [14, 15]. However, of particular note is the fact that our dataset included three TAI cases with a postinjury survival time less than 30 min: a witnessed assault, a witnessed traffic accident, and an unwitnessed simple fall of an elderly person. The postinjury survival estimates were considered to be fairly accurate in these cases. Interestingly, β-APP immunoreactivity has recently been reported among cases with short survival times [16]. Even though our findings appear to align well with this report, we cannot rule out the possibility of previous unknown injuries that may have confounded the interpretation of β-APP positivity in our cases.

Of traumatic findings in autopsy and neuropathology, extracranial, cranial, and intracranial injuries were all associated with TAI over both VAI and no AI. The presence of any cranial/extracranial injury (and each injury type individually) were found to be suggestive of TAI. This was expected due to their role as signs of an external impact to the head. Also the presence of any intracranial injury (and each injury type individually except for epidural haemorrhage) was suggestive of TAI. Our dataset included only one case with epidural haemorrhage, which prevented comparative analyses regarding that variable. In general, intracranial haemorrhages and parenchymal brain injuries are often associated with mechanical tissue damage and thus with AI. For example, traumatic subarachnoid and subdural haemorrhages and contusions have all been associated with AI [17–19].

When VAI and no AI were compared, the presence of cranial/extracranial injuries was found to be suggestive of no AI. It should be noted that TAI cases were not included in these comparisons. Speculatively, the finding may be explained by the fact that these cases involved only external head injuries instead of severe intracranial injuries, or alternatively, these deaths occurred quickly before diagnostic levels of β-APP immunopositivity were cumulated. As for intracranial injuries, the presence of subdural haemorrhage was suggestive of VAI over no AI. This is logical, as primary injuries such as expansive subdural haemorrhage can give rise to secondary complications such as brain herniation [19–21] and thus also VAI. A similar mechanism would be expected to apply also to other intracranial haemorrhages with a mass effect.

Of other neuropathology findings, none were identified as particularly strong at distinguishing between TAI and VAI. This may be due to the fact that our TAI group comprised cases with both “pure TAI” and co-occurring TAI + VAI, and the VAI group comprised cases with only “pure VAI”. When compared to no AI, the presence of brain oedema, herniation, and infarction were indicative of both TAI and VAI. In TAI, the presence of oedema, herniation, and/or infarction are suggestive of a complicated traumatic brain injury. In VAI (without TAI), the presence of oedema, herniation, and/or infarction would point towards alternative non-traumatic aetiologies such as ischaemic stroke [22]; also this entity is encountered relatively frequently in the medico-legal setting.

The strengths of this study lie in a relatively large sample size and a wide variety of background characteristics and autopsy and neuropathology findings that allowed for a versatile analysis of the potential factors associated with TAI, VAI, and absence of AI. All cases were subjected to a comprehensive neuropathological examination performed by a board-certified neuropathologist who also interpreted the β-APP stains, which increases the reliability of the AI diagnostics. Although the interpretation may be difficult in some cases [23], β-APP is widely considered the most reliable method for the diagnosis of AI postmortem [24].

There are also limitations to acknowledge. Even though the diagnoses of TAI and VAI were based on a number of samples obtained from the corpus callosum, internal capsule, and brainstem as the minimum standard, it is possible that focal and unusually located AI findings may have been missed. Of note is the fact that a relatively high percentage of TAI cases had hypoxic-ischaemic neuronal injury (76.9%), though often only mild and terminal, but still possibly suggesting that VAI may have been present in more cases than in which secondary VAI was currently established. Similarly, certain VAI cases were diagnosed with intracranial injuries (18.2%), suggesting that focal TAI may also have been present in these cases. Even though many of our findings were consistent with what is already known about the subject, we report prevalence ratios that demonstrate numerically how likely a finding is in relation to another group in this dataset. As always, a larger sample would have increased the generalizability of our findings, and more detailed information on background characteristics (such as injury mechanisms, timing of injury, and postinjury survival) and neuropathology findings (such as location and severity) would have provided a more fruitful dataset for the analysis.

In conclusion, we report several differences in background characteristics and neuropathology findings between medico-legal autopsy cases diagnosed with TAI, VAI, and no AI. Our findings may help forensic pathologists estimate the likelihood and potential aetiology of AI based on background characteristics and other neuropathology findings.

## Data Availability

The data underlying this article cannot be shared publicly due to local privacy regulations.
